# Old Shoes

**Published:** 1884-02

**Authors:** 


### Old Shoes.

How much a man is like old shoes!
For instance: both a soul may lose;
Both have been tanned; both are made tight
By cobblers; both get left and right;
Both need a mate to be complete,
And both are made to go on feet,
They both need heeling, oft are sold,
And both in time all turn to mold.
With shoes the last is first; with men
The first shall be the last; and when
The shoes wear out they're mended new:
When men wear out they're men-dead, too,
They both are trod upon, and both
Will tread on others, nothing loath.
Both have their ties, and both incline
When polished in the world to shine;
And both peg out—and would you choose
To be a man or be his shoes?

